# Tobacco and ADHD: A Role of MAO-Inhibition in Nicotine Dependence and Alleviation of ADHD Symptoms

**DOI:** 10.3389/fnins.2022.845646

**Published:** 2022-04-12

**Authors:** Mairin Rose Taylor, Kelly Carrasco, Andres Carrasco, Arindam Basu

**Affiliations:** ^1^School of Health Sciences, University of Canterbury, Christchurch, New Zealand; ^2^School of Education, Victoria University of Wellington, Wellington, New Zealand; ^3^School of Health Sciences, Massey University, Wellington, New Zealand

**Keywords:** monoamine-oxidase inhibitors, MAOI’s, ADHD, attention-deficit/hyperactivity disorder, smoking, cigarette, e-cigarette, self-medication

## Abstract

Attention-deficit/hyperactivity disorder (ADHD) is a relatively commonly occurring neurodevelopmental disorder affecting approximately 5% of children and young people. The neurobiological mechanisms of ADHD are proposed to particularly center around increased dopamine receptor availability related to associated symptoms of reduced attention regulation and impulsivity. ADHD is also persistent across the lifespan and associated with a raft of impulsive and health-risk behaviors including substance abuse and smoking. Research highlighting the potentially significant levels of monoamine oxidase (MAO) inhibitory properties in tobacco smoke and e-cigarettes may provide a mechanism for increased tobacco smoke dependence among those with ADHD, in addition to the role of nicotine.

**Aim:**

This scoping review aimed to establish evidence for the above neurobiological pathway between smoking and ADHD symptom-alleviation or “self-medication” with the inclusion of the mechanism of MAO-inhibitors indirect increasing dopamine in the brain.

**Methodology:**

Scoping review methodologies were employed in this review selected to synthesize multiple sources of empirical research to identify current gaps in the knowledge base and identify key characteristics of research data related to a phenomenon. Databases searched included OVID MEDLINE(R), Embase, Cochrane, PsycINFO and SCOPUS limited to 2000 onward and empirically validated, peer-reviewed research.

**Findings:**

There is support for the role of MAO-inhibition on greater reinforcement of smoking for individuals with ADHD through a greater impact on dopaminergic availability than nicotine; potentially moderating ADHD symptoms.

**Conclusion:**

Greater support for a “self-medication” model of ADHD and smoking includes not only nicotine but also MAO-inhibitors as dopamine agonists contained in cigarettes and e-cigarettes.

## Introduction

Attention-deficit/hyperactivity disorder (ADHD) is a relatively commonly occurring neurodevelopmental disorder affecting approximately 5% of children and young people (Sayal et al., 2018). The disorder is relatively pervasive, impacting on comorbidity risk, family functioning, future earnings and other significant health and societal costs (Biederman and Faraone, 2006). Impairment associated with ADHD is persistent across the lifespan (Hammerness et al., 2013) and associated with a raft of impulsive and health-risk behaviors including substance abuse and tobacco smoking (Taylor et al., 2017) and more recently, e-cigarette usage (Xu et al., 2021). Neurocognitive mechanisms of ADHD are proposed to principally center around increased dopamine receptor availability related to associated symptoms of reduced attention regulation (Lou et al., 2004) and impulsivity (Mortimer et al., 2019). Importantly, many adults self-report a high degree of “self-medication” with caffeine and nicotine-containing substances for relief of “core” ADHD symptoms such as restlessness and impulsivity (Bizzarri et al., 2009) and ADHD- *related* symptoms such as sleep and mood functioning (Wilens et al., 2007). Tobacco use is also commonly reported among adolescents with ADHD (Wilens and Kaminski, 2018) and is particularly associated with psychiatric comorbidity (Chang et al., 2012).

Various explanatory models have been proposed in unraveling the relationship between ADHD and nicotine use. These include greater responsiveness among individuals with ADHD to socio-behavioral influences such as imitation and peer pressure (van Amsterdam et al., 2018). An early hypothesis of sensitization to substance abuse due to increased reinforcement from long-term stimulant medication was posited due to animal-models of dopaminergic systems (Goldman et al., 1998). However, as described by Schoenfelder et al. (2014) in their meta-analysis this early model has been readily challenged by several human studies demonstrating significant reductions in nicotine usage relating to long-term stimulant medication (Groenman et al., 2013).

The potential interplay between self-medication models and socio-behavioral influences between ADHD and nicotine have been reviewed by van Amsterdam et al. (2018). The neurocognitive mechanisms described in their review posit the role of aberrant striatal dopaminergic systems in ADHD and the indirect dopamine enhancing effects of nicotine.

This current scoping review seeks to further explore mechanisms of a self-medication model of ADHD and nicotine by including the possibly important role of monoamine oxidase inhibitory (MAOI) activity. Such an inclusion of MAOI activity is timely given the wider usage of e-cigarettes containing physiologically significant levels of MAO inhibitory activity (Truman et al., 2019) and the potentially key role of MAO dysregulation on serotonergic and/or dopaminergic systems (and norepinephrine) in individuals with ADHD (Nikolaus et al., 2021).

## Dopamine Model of Attention-Deficit/Hyperactivity Disorder

Dopamine release has been implicated as a key mechanism of ADHD symptomology. In particular, neurobiological models have postulated that atypical properties of dopamine release impact the reward processing pathway of individuals diagnosed with ADHD (Wu et al., 2012). Neuroimaging studies (Spencer et al., 2007) and research into the genetics of ADHD (Durston and Konrad, 2007) have evidenced atypical dopamine transporter in the striatum of adults with ADHD that may result in underactivity in dopaminergic pathways. While stimulant medications, such as methylphenidate, alleviate symptoms of ADHD by altering dopamine release properties (Storebø et al., 2015), the complete neurobiological basis of ADHD remains poorly understood.

The dopamine transfer deficit (DTD) model proposed by Tripp and Wickens (2008) is a leading neurobiological framework of ADHD. In this model, temporal variations in dopamine release provoked by environmental signals are postulated to induce ADHD behavioral symptoms. This model proposes that dopaminergic neuronal responses to positive reinforcement transfers to preceding neutral cues in typically developing (TD) individuals, but not in people with ADHD. Therefore, individuals with ADHD lack the dopamine signalling to the anticipatory cue of reinforcement. Thus, individuals with ADHD will display a more rapid behavioral extinction if reinforcement is delayed or discontinued considering the lack of anticipatory dopamine signalling from the cue. This framework provides an explanatory model of some core symptoms of ADHD, including a lack of inhibitory control for immediate rewards and a delay aversion to larger rewards (Coghill et al., 2014). The role of dopamine in ADHD is well-evidenced and therefore any neurobiological model of “self-medication” among individuals with ADHD would likely include dopamine as a key mechanism.

## Method Section

A scoping methodology has been selected for this review as it follows the suggestions of Peters et al. (2015) to synthesize multiple sources of empirical research in order to identify current gaps in the knowledge base and identify key characteristics of research data related to a phenomenon (Arksey and O’Malley, 2005; Munn et al., 2018). As scoping reviews aim to present a broad overview rather than a critical synthesis of data, an assessment of methodological limitations or bias is not included in this review as supported by Munn et al. (2018).

### Review Question/Objective

This review seeks to further explore mechanisms of a self-medication model of ADHD and nicotine by including the possibly important role of monoamine oxidase inhibitory (MAOI).

1. Is there evidence for the role of monoamine oxidase inhibitory (MAOI) compounds influencing aberrant striatal dopaminergic systems in ADHD and dopamine enhancing effects of cigarettes (e-cigarettes, vapes)?

### Search Procedure

Studies were identified using OVID MEDLINE(R), Embase, Cochrane, PsycINFO and SCOPUS searches. Boolean operators were employed with terms such as: “nicotine, cigarette, smoking tobacco, vaping, e-cigarette; ADHD, attention-deficit, hyperkinetic and dopamine, monoamine oxidase, MAO, or MAO-inhibition.”

### Eligibility Criteria

Both animal and human research studies were included. Randomized or non-randomized studies were included, as well as prospective or retrospective naturalistic studies.

### Publication

In order to maintain current relevancy, the time period of focus was 2000 onward in order to build on the growing resource of reviews of research and literature completed since 2000. Exceptions to this time-frame included studies that were foundational to the topic. Empirically-validated, peer-reviewed research were the focus. Gray research such as policy papers and evaluative research were excluded.

### Data

To be included in the analysis studies must have included information regarding effect size (or other analyses demonstrating strength of relationship between variables) and information relating to methodologies employed. Studies were excluded due to lack of outcome measures or insufficient data relating to methodology.

### Data Extraction and Charting the Results

**FIGURE 1:**
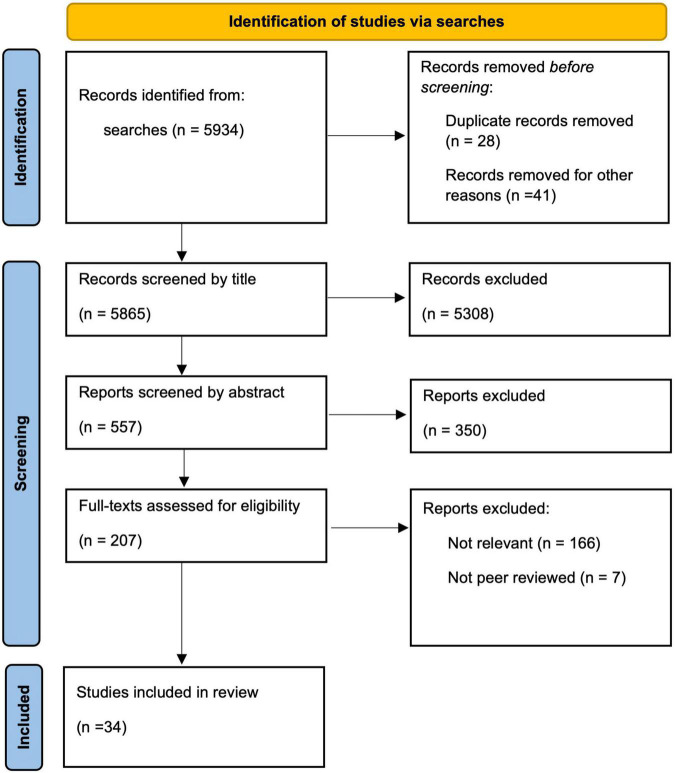
PRISMA flow diagram for the scoping review process goes here.

Eligible studies (*n* = 5934) were initially screened by one author through review of titles and if needed abstracts, to ascertain pertinence to the subject area. The same author reviewed the abstracts of the remaining studies (*n* = 557) to extract methodological information and statistical information. These studies were then reviewed by two authors to assess eligibility from abstracts and full-text articles for the review of strength of evidence (*n* = 34). See Figure 1 for the Preferred Reporting Items for Systematic Reviews and Meta-Analyses (PRISMA) flow diagram for the scoping review process (Page et al., 2021).

## Results

**FIGURE 2:**
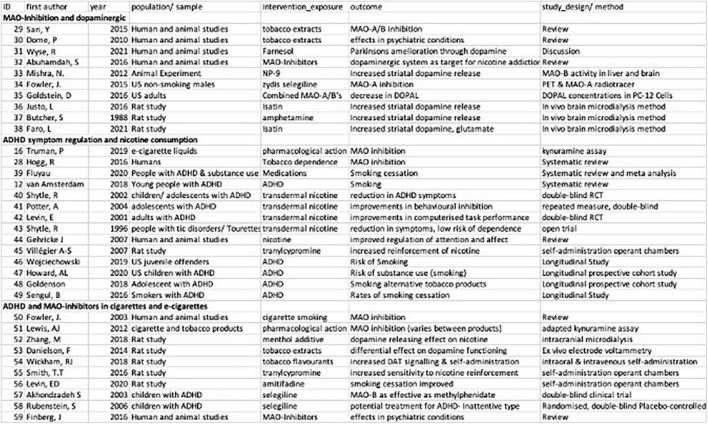
Summary of studies included in scoping review of MAOI- related self-medication models of ADHD and nicotine.

Well established correlations in the self-medication model of ADHD are previously described in the introduction of this review, including the role of dopaminergic-dysregulation and ADHD (Wu et al., 2012; Nikolaus et al., 2021) and the presence of MAO-inhibitory compounds in cigarettes, e-cigarettes and vape fluid (Hogg, 2016; Truman et al., 2019). Instead, the following results are focused on a descriptive review of the three focus areas of this scoping review: MAO-Inhibition and dopaminergic regulation, ADHD symptom regulation and nicotine consumption and ADHD and MAO-inhibitors in cigarettes and e-cigarettes (see Figure 2).

### Monoamine Oxidase-Inhibition and Dopaminergic Regulation

An early review of MAO-A/B inhibitory compounds in tobacco smoke identified several compounds that are reversible and either selective or non-selective inhibitors, including 2,3,6-trimethyl- 1,4-naphthoquinone; 2-naphthylamine; norharman; harman; farnesol, and farnesylacetone (Sari and Khalil, 2015). While there has been some discussion of the potential role of these MAO-inhibiting substances on diseases such as Parkinson’s (Dome et al., 2010; Wyse et al., 2021) *via* changes in dopaminergic systems (Abuhamdah et al., 2016); there have been few studies to date that demonstrate reliable cognitive effects of those specific compounds found in tobacco products.

Rat model studies largely focused on dopaminergic models of Parkinson’s disease have relevance for understanding the general *mechanisms* of MAO-inhibition and dopaminergic systems. For example, Mishra and Sasmal have demonstrated the *direct* effect of chronic exposure to a MAO-B inhibitor on an increase in striatal dopamine levels (Mishra and Sasmal, 2012). The authors also noted behavioral changes in observed rats including potentiating induced stereotyped movements and ameliorating oral dyskinesia (of import to dopaminergic models of Parkinson’s disease).

Fowler et al. (2015) tested the role of an MAO-B inhibitor (selegiline), a Parkinson’s disease treatment, on MAO-A inhibition. Adult males in this study were found to have “co-inhibited” MAO-A in addition to MAO-B inhibition. Goldstein et al. (2016) then related MAO-A/B inhibition (including selegiline) to the decrease in production of dopamine metabolite 3,4-dihydroxyphenylacetaldehyde (DOPAL) in rats, thereby increasing the availability and reducing the synthesis/re-uptake of dopamine. Relatedly, Justo et al. (2016) found an infusion of isatin (an MAO- A/B inhibitor) increases dopamine release in rats. The authors suggested in agreement with others, that isatin may increase dopamine levels through greater availability of dopamine in the cytoplasmic reservoir (Butcher et al., 1988; Justo et al., 2016). Isatin was further investigated by Faro et al. (2021) who found that this MAO-inhibitor likely increases dopamine availability through a) suppression of dopamine metabolism, and b) increasing dopamine release by stimulating exocytotic dopamine production. In summary, there is emerging and convincing evidence of MAO-inhibitors directly and indirectly increasing dopamine in both animal and human models.

### Attention-Deficit/Hyperactivity Disorder Symptom Regulation and Nicotine Consumption

Attention-deficit/hyperactivity disorder is associated with increased risk of smoking initiation at an early age, maintenance of smoking, and reduced propensity for smoking cessation for adults, possibly mediated by dopamine receptor activity patterns, in turn mediated by MAO-inhibitory contents and nicotine in cigarettes and e-cigarettes (Hogg, 2016; Truman et al., 2019). We found evidence in support of whether *nicotine* consumption in doses compatible with smoking, alleviates ADHD- related symptoms. Stronger evidence was found in support of the role of *MAO-inhibiting* compounds also found in cigarettes, e-cigarettes/vape fluid.

We identified eight twelve studies on the relationship between ADHD and smoking or nicotine replacement therapies. Fluyau et al. (2021) conducted a systematic review of treatment of substance use disorders among ADHD patients where they evaluated the overall effectiveness of interventions and therapies targeted at a reduction of both substance abuse but also reduction in the symptom severity of ADHD. In the subset of candidate studies on smoking cessation, they found that: the nicotine antagonist in brain (Varenicline, RR: 0.76, 95% CI: 0.49–1.05), nicotine patch with counseling and methylphenidate treatment all were effective in the treatment of both ADHD but also led to smoking cessation and abstinence among smokers.

The systematic review by Fluyau et al. (2021) aimed to study whether concurrent pharmacological treatment of substance use and ADHD were beneficial for both phenomena. While a single metric was not reported in their study, the findings suggest that when both ADHD symptom control and smoking cessation were targeted with the same medication, in general, while ADHD symptoms were ameliorated, smoking cessation was not achieved or the results were equivocal (Fluyau et al., 2021). van Amsterdam et al. (2018) in their systematic review tested the self-medication hypothesis. According to the “self-medication hypothesis,” people with ADHD tend to initiate and continue smoking because smoking provides them with a supply of nicotine that aims to supplement deficient dopamine in their cortical-striatal pathways leading to alleviation of symptoms. van Amsterdam noted in their review, mixed evidence in support of this self-medication hypothesis as some studies that included nicotine analogs varenicline, and bupropion did indeed improve symptoms of ADHD and also resulted in better abstinence and less risk of smoking initiation. However, they concluded that evidence in favor of self-medication hypothesis was inconclusive and needed verification based on population based longitudinal studies (van Amsterdam et al., 2018). In addition, while studies that have employed transdermal nicotine administration have been shown to reduce ADHD symptoms in children (Shytle et al., 2002), adolescents (Potter and Newhouse, 2004), and adults (Levin et al., 2001), the side effects as a result of transdermal nicotine have resulted in little therapeutic use in younger populations (Shytle et al., 2002). Considering that nicotine product abuse or initiation of tobacco use has not been shown following transdermal nicotine use in non-smokers with ADHD or the highly related Tourette’s syndrome (Shytle et al., 1996, 2002), Gehricke et al. (2007) suggest that focusing on nicotine alone to study tobacco smoking addiction in individuals with ADHD is restrictive and may be contributing to mixed evidence of the self-medication hypothesis. Rats, for example, have been shown to increase the self-administration of nicotine when given MAOIs, where the inhibition of MAO appears to increase the reinforcing effect of nicotine (Villégier et al., 2007). Taken together, the additional properties of tobacco smoking not found in nicotine products is a likely contributing factor to the maintenance of smoking in those with ADHD.

Wojciechowski (2020) in their analysis of longitudinal data with juvenile offenders with ADHD in the United States found that those with more symptomatology were more likely to be chronic smokers. In their analysis of data from the Multimodal Treatment Study of the Adolescents (MTA) in the United States, Howard et al. (2020) found that among those adolescents who had initiated early smoking, adolescents with ADHD were about three times likely to sustain smoking than those without ADHD (RR: 2.7). The authors noted that early onset smoking was a mediator for smoking later in life and the continuation of smoking for those with ADHD. Goldenson et al. (2018) conducted a longitudinal study with ninth grade US students with ADHD who were non-smokers reported that those with higher scores on ADHD were more likely to later report e-cigarette, hookah and cigarette smoking. In a study on Turkish adult smokers, Şengül et al. (2016) reported that those with ADHD symptoms had a higher failure rate for smoking cessation (OR: 2.12; 95% CI: 1.02–4.40). Taken together, the body of evidence indicate that ADHD both predisposes individuals to higher risks of smoking or nicotine dependence, and likewise, higher risks of failure to quit.

### Attention-Deficit/Hyperactivity Disorder and Monoamine Oxidase-Inhibitors in Cigarettes and E-Cigarettes

We identified studies that potentially “fill the gap” identified by van Amsterdam et al. (2018) as MAO-inhibitors contained in cigarettes and e-cigarettes are not attributed to nicotine itself (Fowler et al., 2003). In Hogg’s review of tobacco smoke derived MAO-inhibition, the author found strong evidence for MAO-inhibition from substance/s in or derived from non-nicotinic tobacco smoke (Hogg, 2016). Likewise, Lewis et al. (2012) found that ‘roll-your-own tobacco products tend to deliver more MAO-inhibitory compounds than commercial varieties of cigarettes and Zhang et al. (2018) found a dopamine-releasing effect of menthol on nicotine (Zhang et al., 2018). In keeping with changes in smoking delivery methods, Truman et al. (2019) have identified moderate to high levels of MAO-A/B inhibitory activity in certain e-cigarette flavors.

Importantly, a differential effect of nicotine versus tobacco extract on dopaminergic systems was found by Danielson et al. (2014) in which dopamine increases were identified following tobacco delivery but not nicotine. Wickham et al. (2018) too found that tobacco product flavor additives increase dopamine (DA) signalling and increase self-administration behavior. The role of MAO-inhibitors on smoking cessation is also likely to have been historically under-scrutinized. MAO-inhibitors may increase the reinforcing value of low doses of nicotine (Smith et al., 2016) and Levin et al. (2020) found that the joint administration of nicotine with amitifadine, a triple monoamine reuptake inhibitor, greatly aids smoking cessation (Levin et al., 2020). All studies together support the role of both tobacco smoke compounds and e-cigarette flavourants in MAO-inhibition and dopaminergic systems.

Intervention research further adds support for the important role of MAO-inhibition on ADHD- symptom control. Among individuals with ADHD, the effective treatment of ADHD with MAO-inhibitors such as selegiline (Akhondzadeh et al., 2003; Rubinstein et al., 2006) are found to have similar effectiveness as methylphenidate, as reviewed by Finberg and Rabey (2016). The studies reviewed add weight to the hypothesis that adults with ADHD may be at a greater vulnerability to cigarette and e-cigarette dependence potentially mediated by MAO-inhibitory compounds that may influence dopaminergic systems.

## Discussion

Our review of evidence supports the finding that individuals with ADHD are at greater vulnerability for both initiation and continuation of smoking (both cigarettes, e-cigarettes) (Goldenson et al., 2018). This is further supported by a large study of Italian adolescents in which cigarette smoking was associated with high levels of impulsivity for both males and females (Di Nicola et al., 2017). A previous review conducted by van Amsterdam et al. (2018) explored the “self-medication” hypothesis of ADHD and nicotine dependence, a logical explanatory model of this vulnerability. However, the evidence for this hypothesis was found by the authors to be inconclusive. One of the key limitations to the self-medication hypothesis is that long-term methylphenidate treatment for ADHD has been found in some studies to reduce nicotine dependence (Schoenfelder et al., 2014) but not so in others (Humphreys et al., 2013). However, this mechanistic pathway is dependent on “dopaminergic medications… acting as a substitute for nicotine” (van Amsterdam et al., 2018, p.436). The authors of this current scoping review sought to explore whether nicotine alone may not be a *sufficient* mechanism for the relationship between ADHD + dopaminergic dysregulation in smoking initiation and dependence. The important role of MAO-inhibitors is supported by evidence suggesting that MAO-B is inhibited in the brains of smokers in the general population, thereby increasing dopamine availability (Fowler et al., 1996) and that the joint administration MAO-inhibitors, greatly aid smoking cessation (Smith et al., 2016; Levin et al., 2020).

The above scoping review demonstrated promising evidence for the additional (or even primary) role of MAO-inhibitory compounds in cigarettes and e-cigarettes in greater vulnerability to smoking abuse and dependence among individuals with ADHD. This hypothesis is based on dual factors: evidence to suggest that MAO-inhibitors in cigarettes and e-cigarettes may have a stronger effect on dopaminergic systems than nicotine (Danielson et al., 2014) and that MAO-inhibitors may have an ADHD symptom alleviating effect at a level commensurate with stimulants (Finberg and Rabey, 2016) presumably similarly modifying the dopaminergic pathway (Mishra and Sasmal, 2012).

A well-addressed limitation of scoping reviews is the lack of clarity around strength of evidence (Munn et al., 2018). Furthermore, there is to date limited evidence on the comparative treatment of smoking in adults with ADHD with MAO-inhibitors and limited animal research that evidences the mechanism of MAO-inhibitors in increasing dopamine availability in low attention/high impulsivity rats. However, this review does suggest there is merit in future systematic reviews of the role of MAO-inhibitors as a mechanism for ADHD- related smoking dependence. There are clinical implications to this study as the investigation of psychopathological vulnerabilities that may underly addictive behaviors may be crucial to building preventive measures and the delivery of targeted interventions for vulnerable populations, such as adolescents with ADHD.

## Author Contributions

All authors listed have made a substantial, direct, and intellectual contribution to the work and approved it for publication.

## Conflict of Interest

The authors declare that the research was conducted in the absence of any commercial or financial relationships that could be construed as a potential conflict of interest.

## Publisher’s Note

All claims expressed in this article are solely those of the authors and do not necessarily represent those of their affiliated organizations, or those of the publisher, the editors and the reviewers. Any product that may be evaluated in this article, or claim that may be made by its manufacturer, is not guaranteed or endorsed by the publisher.
